# The Evolution of Orbital Implants and Current Breakthroughs in Material Design, Selection, Characterization, and Clinical Use

**DOI:** 10.3389/fbioe.2021.800998

**Published:** 2022-02-17

**Authors:** Xiao-Yi Chen, Xue Yang, Xing-Li Fan

**Affiliations:** ^1^ Plastic and Reconstructive Surgery Center, Department of Plastic and Reconstructive Surgery, Zhejiang Provincial People’s Hospital, Affiliated People’s Hospital of Hangzhou Medical College, Hangzhou, China; ^2^ Key Laboratory of Tumor Molecular Diagnosis and Individualized Medicine of Zhejiang Province, Zhejiang Provincial People’s Hospital, Affiliated People’s Hospital of Hangzhou Medical College, Hangzhou, China; ^3^ Clinical Research Institute, Zhejiang Provincial People’s Hospital, Affiliated People’s Hospital of Hangzhou Medical College, Hangzhou, China; ^4^ School of Basic Medicine and Forensic Medicine, Hangzhou Medical College, Hangzhou, China

**Keywords:** orbital implants, biomaterial, ophthalmology, material design, clinical translation

## Abstract

It is occasionally essential to surgically remove the damaged eye of the patient in the case of serious oculoorbital injuries, intraocular cancers, and other life-threatening diseases. An orbital implant is placed into the anophthalmic socket after the eye is removed to provide adequate volume reinstatement and revamp the cosmetic look of a normal eye. In the previous few decades, implant design and material selection criteria have progressed from basic nonporous polymeric spheres to devices with more complicated shapes and functions to ensure improved long-term clinical results. Because of their highly interconnected porous design, ceramic and polymeric porous implants have found popularity as a passive framework for fibrovascular ingrowth, with lower obstacle rates and the option of setting to improve prosthetic eye mobility. These materials, however, are not without flaws. The danger of migration and extrusion, infections after surgery, and poor motility transferred to the cosmetic ocular prosthesis are important elements of orbital implants of today. As a result, the development of novel biomaterials with improved functionalities (i.e., antibacterial effect, angiogenesis, and *in situ* moldability) that allow better eye replacement is more desirable than ever, highlighting one of the most challenging aspects of research topics in the field of ocular implants. This study highlights the history of orbital implants. It gives an outline of current advancements in the area, over and above some essential observations for materials design, selection, characterization, and transformation to clinical applications.

## Introduction

**FIGURE 1:**
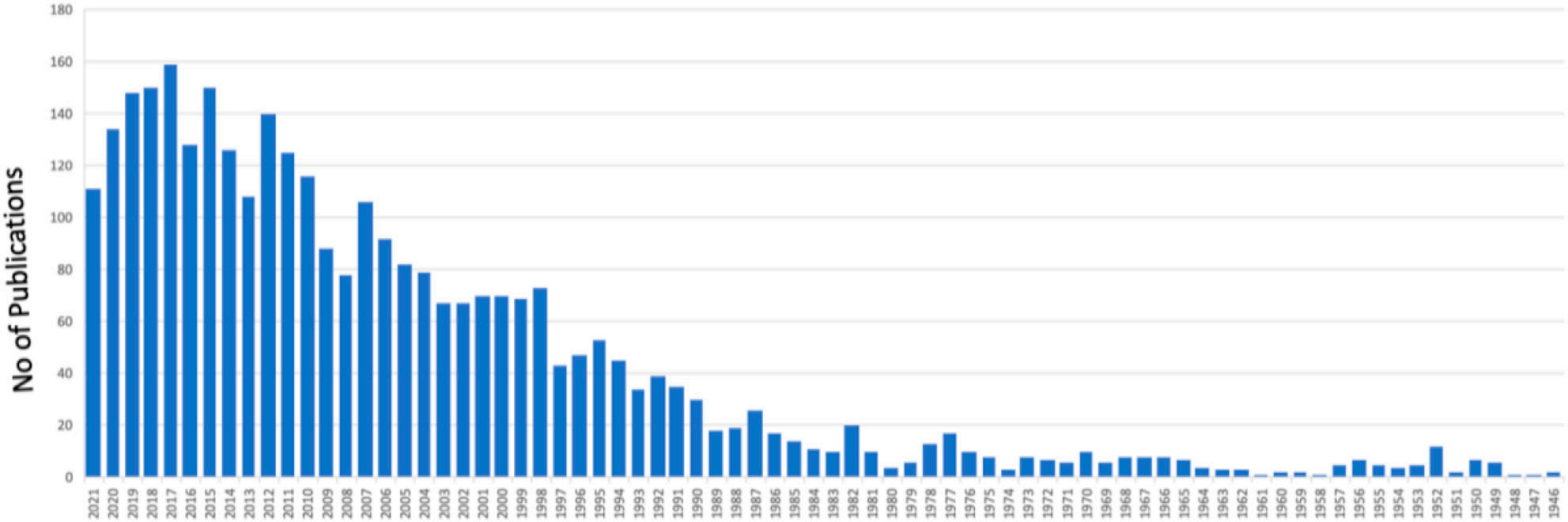
A graph shows the number of publications in “orbital implants” from 1946 to date (PubMed in August 2021).

Since the first coralline hydroxyapatite porous orbital implant was introduced in the early 1980s for eye replacement, various more modified porous implants have been produced (Cleres and Meyer-Rüsenberg, 2014). In cases of different circumstances, untreatable, frequently serious illnesses affecting the oculoorbital structures of the patient, a surgeon must propose the removal of an eye (Saxby et al., 2019; Pine et al., 2011). There are various reasons to consider this extreme treatment, including irreversible eye injury from trauma, severe intraocular infection, and malignant intraorbital tumors or agonizing blindness (Baino and Potestio, 2016). Surgical removal of orbital soft tissue subjects should be accomplished in three ways, reliant on the pathophysiology of the individual patient (Moshfeghi et al., 2000). In the last 2 decades, orbital implantation has been rapidly increasing, as manifested by the increasing research (Figure 1). Evisceration is a surgical procedure that involves removing the viscera (uvea) of the eye while leaving the extraocular muscles, Tenon’s capsule, scleral coat, and optic nerve intact. It is usually performed in a sightless and/or aching eye with no helpful optical potential because of a serious intraocular infection. Enucleation is the surgical removal of the entire eyeball by severing the optic nerve near the earth and cutting the extraocular muscles; the most common reasons for enucleation are irreversible oculoorbital injuries and malignant cancers. Exenteration is the most invasive of the three operations, and it is the sole option for certainly treating the most advanced malignant cancers in the path; it entails eradicating the entire orbital substances down to the bone. Continuous advancements in microsurgery and medicinal treatments have led to a decline in the general mean yearly prevalence of enucleations over the past 25 years, while the occurrence of serious ocular trauma and ocular cancer (frequently inherited) has remained relatively steady (Geirsdottir et al., 2014). An orbital implant is placed after evisceration or enucleation to restore the missing orbital volume. The extraocular muscles stay connected to the scleral wrapper that ranks the graft in evisceration surgery. However, in enucleation surgery, the muscles must be reattached either directly to the implant (if flexible and malleable) or indirectly to a wrapping material over the implant (Sami et al., 2007; Baino et al., 2014). A custom-made prosthesis that rebuilds substantial portions of the orbit and even the face skin are routinely inserted in exenterated patients. The surgeon determines the size of the orbital implant during surgery, and it is established on the anatomic demands of every individual patient (Adams et al., 2014; Mourits et al., 2015). It is best to choose an implant to replace 65%–75% of the original ocular globe volume (Hughes, 2007).

**FIGURE 2:**
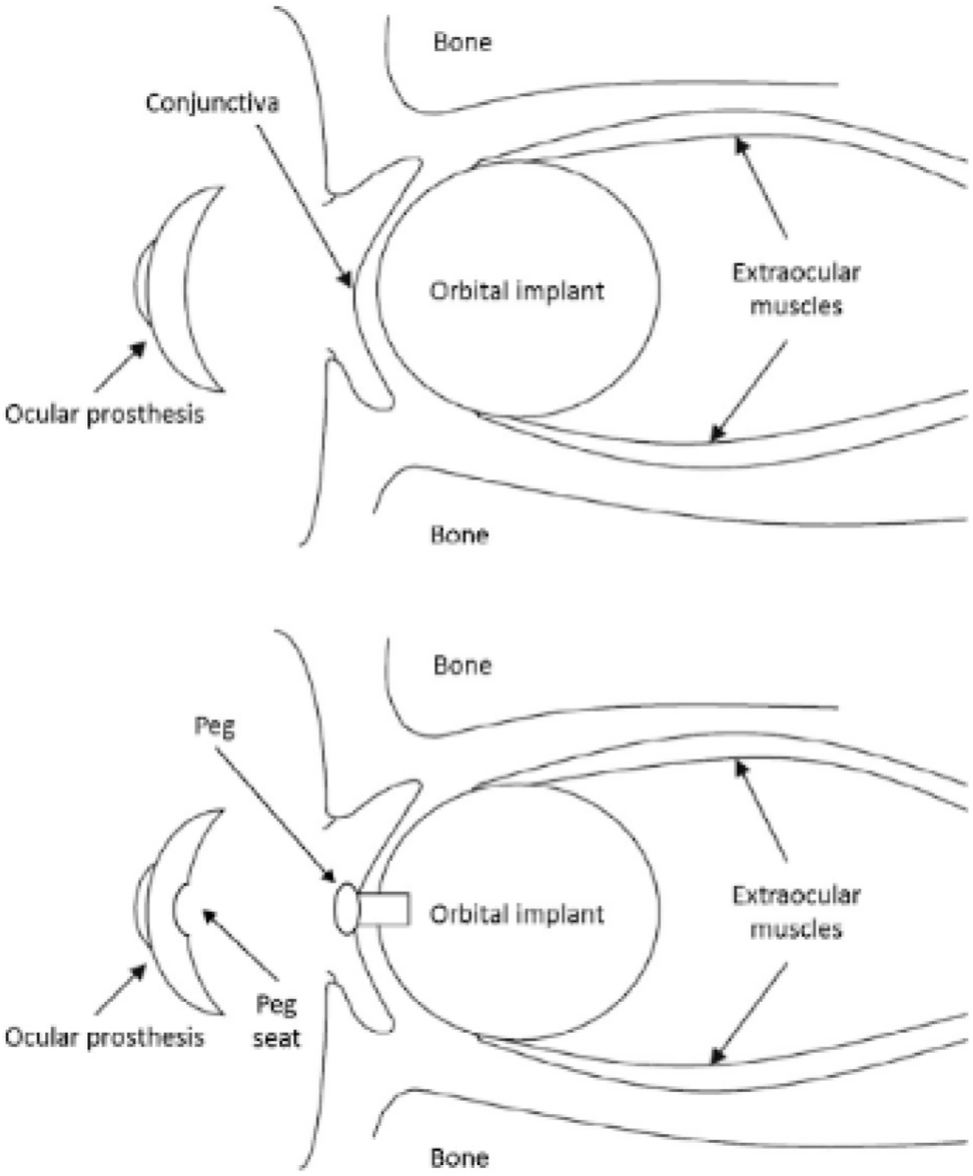
Images of a human orbit following enucleation surgery and spherical implant implantation. Extraocular muscles are sutured directly to the implant in these photos. Reproduced with permission (Baino et al., 2014).

A (aesthetic) visual prosthesis, which fits above the graft and rests just beyond the eyelids, and every draping material utilized to conceal the implant replace the remaining volume. To allow for tissue recovery and suture absorption, the prosthesis is usually not fitted till 6–8 weeks following surgery. Artificial eyes constructed of polymethyl methacrylate (PMMA) have been widely used; meanwhile, in the emergence of acrylic polymers in 1940s, formerly World War II, the visual prosthesis was prepared from glass; however, they had to be worn with caution because of their extreme brittleness. PMMA visual prosthesis are often custom-made devices that exactly match the contours of the orbital tissues and replicate the cosmetic aspects of the contralateral healthy eye (e.g., iris color) (Sethi et al., 2014), although low-cost stock prostheses are also accessible (Geirsdottir et al., 2014). If linking with an orbital implant is not possible due to cost, adhesive-retained silicone ocular prosthesis may be an alternative (Hughes, 2007). Modern enucleation methods, especially the meticulous connection of extraocular muscles to the implant, really equal evisceration in preserving artificial eye movement and aesthetic results. After evisceration or enucleation, an orbital implant is inserted within the scleral envelope, and the patient wears an ocular prosthesis to restore an appropriate cosmetic look (Figure 2).

Early problems (those happening in 6 months of operation) and late difficulties (those taking place in 6 months as soon as implant placement or beyond) succeeding anophthalmic orbit renovation are the consequence of both material and procedure correlated variables (Chalasani et al., 2007).

**FIGURE 3:**
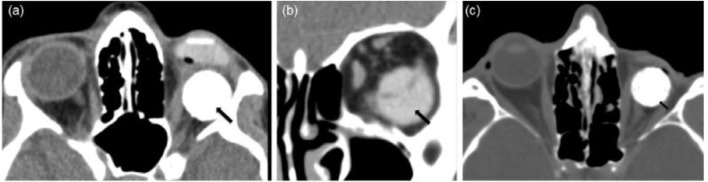
The images are examples of orbital implant complications: **(A)** axial and **(B)** coronal CT images displaying an orbital prosthetic implant that has relocated inferotemporal (black arrows) whereas the underlying ocular prosthesis is properly sited; **(C)** axial CT image illustrating the “postenucleation socket syndrome”—decreased orbital volume on the left with the posteriorly placed hydroxyapatite spherical implant; and **(D)** (black arrow). Reproduced with permission from (Adams et al., 2014).

The “ideal one” of choice among all possible implants is presently a point of contention since each type of implant has both merits and disadvantages. All implants are still susceptible to migration/extrusion and subsequent infection, necessitating more studies to enhance the clinical results of eye replacement. As shown by the growing sum of research papers in the area directly above the previous few decennaries, this review paper was produced in reaction to the increased attention, advancement, and study effort in designing efficient orbital implants (Figure 3). A review of enucleation implants was published recently (Baino et al., 2014), although it is still inadequate, especially in light of recent developments. This evaluation now provides an up-to-date picture, including the most recent results as well as a prognosis for the future. Additionally, towards the end of the study, certain operational observations on the creation and analysis of orbital implant materials and an overview of patenting concerns are offered to encourage debate among researchers working on the subject.

## An Overview of the Various Biomaterials for Ocular Implants

### Autologous Materials

Orbital implants (OIs) are often made of artificial materials (such as ceramics and polymers); but, in rare situations, utilizing autologous ingredients to restore orbit space may be better. When it comes to adult enucleation (human-made implants are more costly), this method is frequently driven by economic considerations, or it may be favored in the juvenile persons, whose tissues and skeletal arrangements will develop and alter throughout time (Hauck and Steele, 2015). Dermal fat grafts are suggested in this regard for their development possibility (Heher et al., 1998) in primary and secondary enucleation (Nentwich et al., 2014); however, graft absorption is frequently unexpected and reliant on the vascularity of the beneficiary bed, which should be impaired following operation and radiation (with regard to ocular cancers) (Raizada et al., 2008). Postauricular skin graft (Wei and Liao, 2014) and cancellous and cortical bone grafts having musculus temporalis flap (Habal, 1987) and anterolateral thigh flaps (Hynes et al., 2016) are further described alternatives. Dermis fat implant grafting in the orbit is also indicated when the patient cannot bear the existence of an artificial material in the anophthalmic socket (untreatable long-lasting pain, permanent inflammation) (Shams et al., 2015). The usage of a periumbilical fat autograft in conjunction with a tiny dripping orbital graft for socket volume escalation following enucleation has been documented to decrease implant exposure (Medel et al., 2016).

### Polymeric Grafts

**FIGURE 4:**
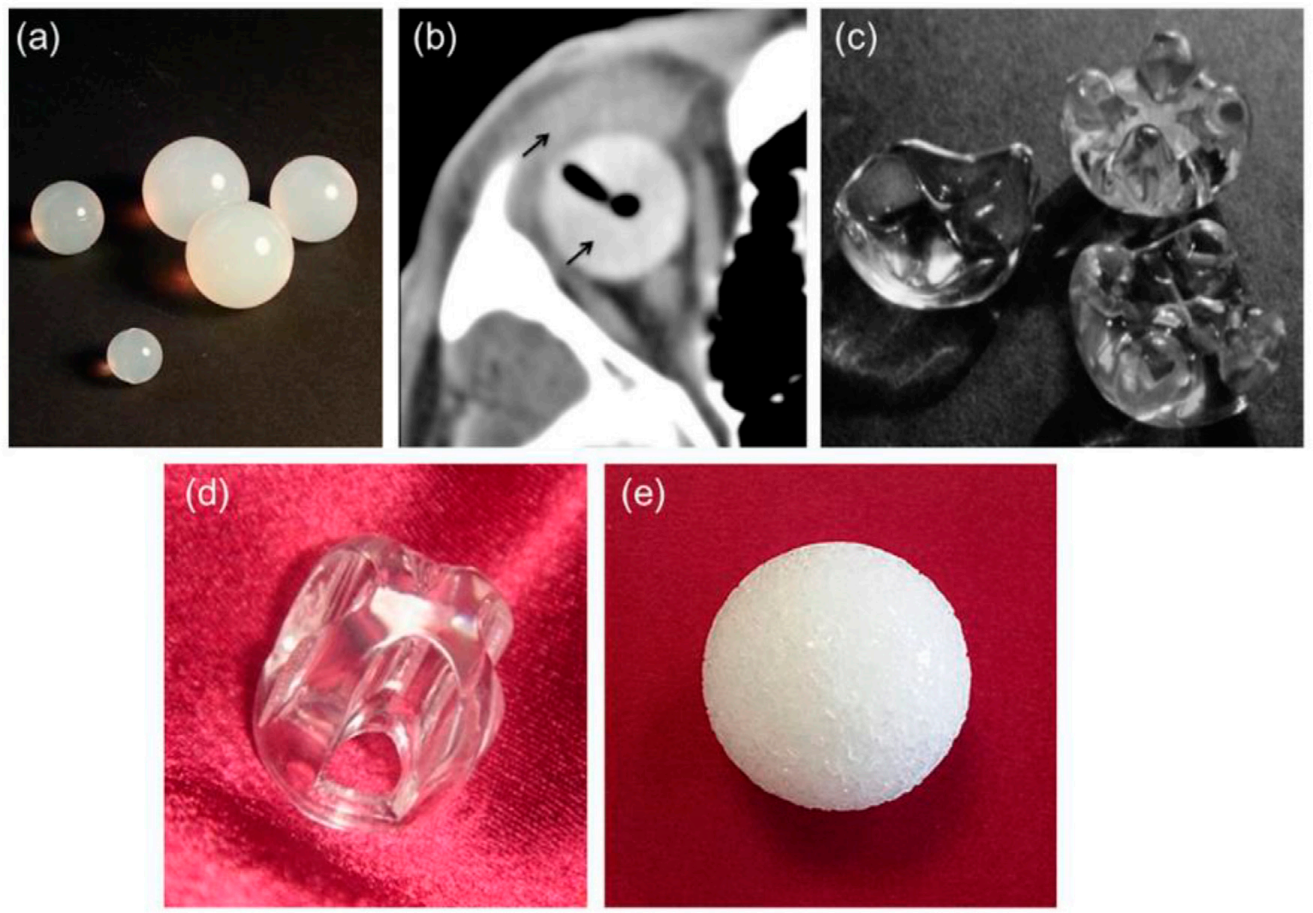
**(A)** silicone spheres, **(B)** axial CT image of an acrylic spherical implant with ocular prosthesis implanted *in vivo* (thin black arrows), **(C)** acrylic orbital implants of the “Allen family” (Iowa implant and its conformer—upper right corner and left, respectively; universal implant—lower right; the size of the implants around 20 mm 20 mm), **(D)** Castroviejo implant, and **(E)** porous sphere. Reproduced with permission from (Adams et al., 2014).

Polymeric orbital implants (PMOI) first appeared after the 2nd World War and are still used extensively, owing to their inexpensive cost related to different options (i.e., ceramic porous implants) for the reason of their well-established biocompatibility inertness and relative pliability (McGregor, 1954). Silicone is widely utilized to manufacture sphere-shaped nonporous orbital grafts (Figure 4A), making it a very appropriate substance for ophthalmic implants (Baino, 2010; Baino, 2011a; Baino, 2011b).

The problematic rate of silicone orbital grafts is typically minimal (Nunery et al., 1993a; Nunery et al., 1993b), although one documented disadvantage is the development of a thick, avascular fibrous capsule around the graft (Sami et al., 2007). Due to the ease of removal for subsequent implant exchange, surgeons appear to favor using a nonporous silicone implant when repairing an anophthalmic socket in a juvenile patient (Piest and Welsh, 2002). Because of its high biocompatibility, PMMA is another widely utilized polymer in ophthalmic applications; it is now the most often used polymeric biomaterial to produce intraocular lenses and hard contact lenses (Bozukova et al., 2010) as well as OIs. Nonporous PMMA spheres (Figure 4B) can be used in primary and secondary (or “definitive”) OIs (Tyers and Collin, 1985; Leatherbarrow et al., 1994) and are still widely used because of their inexpensiveness, simplicity of surgical insertion, and usually positive therapeutic results (Mourits et al., 2016). This novel implant (the Iowa implant) was made by injecting methyl methacrylate resin into four peripheral mounts on the frontal surface corresponding to four similar dejections on the subsequent prosthetic surface (Figure 4C). The mounds provide two positions, making it easy to suture the horizontal and vertical muscle stumps together. Retrospective investigations revealed that these types of implants have low exposure and extrusion rates; nonetheless, those that fail are most frequently due to necrosis of tissues above the mounds (Spivey et al., 1969). In response to this issue, a modification of the Iowa implant was created in 1987 and is now recognized as the universal implant (UI) (Figure 4D). The projecting mounds on this maneuver are reduced and more spherical, possibly reducing problems, however maintaining the motility benefits of the Iowa implant. Even if porous materials have become more popular in recent years, the UI is still a viable option for achieving good artificial eye motility (Klapper et al., 2003). The Castroviejo implant, which acts as a flat-convex fundamental surface above which the synthetic eye may glide, is a variant of the Allen-type devices that adds mobility to the ocular prosthesis (Lee et al., 2000). It features a central dejection encircled by four channels on the front; the four rectimuscles are housed in channels right below the bridge. The opposing ends of the muscles are stitched together to overlap (Figure 3D). Under the conjunctiva, the implant is entirely hidden. In Pakistan (Siddiqi et al., 2008), where the UI and porous orbital implants must be purchased overseas at a heavy price and with a lengthy waiting period, an affordable variant of the Allen-type implant (also called Sahaf implant type I) was created and medically utilized. In Pakistan, a pear-shaped nonporous PMMA graft enfolded in donor sclera or autogenous fascia lata was used for the exenteration and enucleation operations with good results (Kamal et al., 2010). Specific PMMA maneuvers have been created as “auxiliary implants to alleviate particular post-enucleation adverse effects.” The Codere–Durette graft, for example, is a smooth acrylic plate with a horizontal “hill” that may both repair an orbital floor fracture and correct for a superior sulcus deformity of the anophthalmic socket (Dresner et al., 1991). PE in its high concentration form, which can survive sterilizing temperatures, is another polymeric material that has risen in favor of the production of OI (Karesh and Dresner, 1994). Unlike most other polymeric orbital implants, PE devices feature a network of linked holes that permit tissue to grow in. The so-called “Medpor implant,” an inexpensive substitute for ceramic porous implants, is a good commercial example. The Medpor material, which is produced by molding medical-grade high-density PE granules into a spherical form, obtained FDA clearance for use in oculoplastics in 1985 (Karesh and Dresner, 1994) (Figure 4E). Orbital soft tissues usually tolerate porous PE implants well, and they have a plane surface that prevents annoyance of the overlaying conjunctiva after insertion, lowering the likelihood of postoperative problems [foreign body response to absorbent PE is particularly infrequent (Timoney et al., 2016)].

### Ceramic implant

**FIGURE 5:**
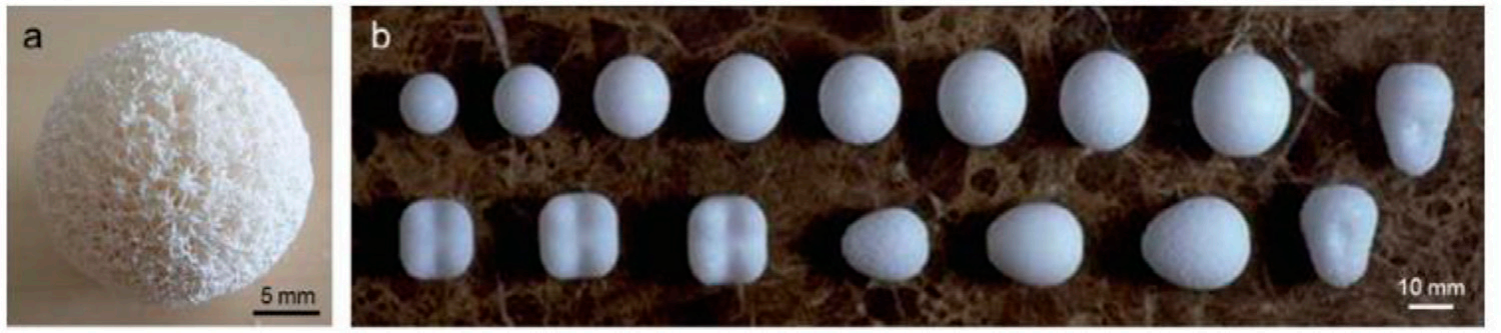
Example of porous orbitals: **(A)** coralline HA sphere and **(B)** various porous PE implants. Reproduced with permission from (Jordan et al., 2010).

The first orbital implant of Mules [a hollow glass sphere (Mules, 1885)] was ceramic since glass is a noncrystalline oxide-built material. Despite their extreme brittleness, these grafts were the standard until the 1940s (Culler, 1952). Some examples of porous orbital are shown in Figure 5.

**FIGURE 6:**
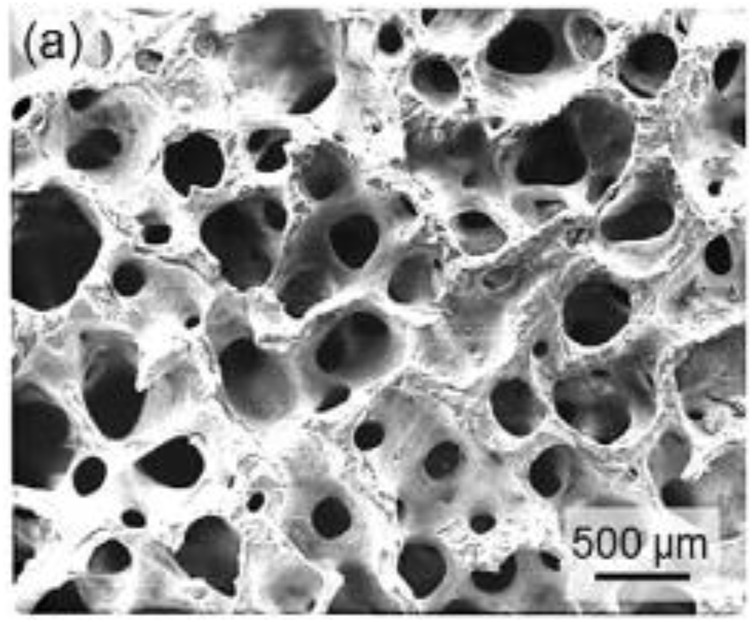
Ceramic orbital implants: **(A)** SEM micrograph of a Molteno M-Sphere. Reproduced with permission.

However, in present years, the use of glass to formulate OI has just about completely disappeared—it has been used in a few rare situations where patients could not tolerate alternative ceramic or polymeric biomaterials and autografts were not an option (Helms et al., 1987; Christmas et al., 1998). In recent decades, porous ceramic implants have grown in popularity because their densely interrelated orifice network permits them to function as a passive structure for host fibrovascular ingrowth, resulting in low problematic rates and improved prosthesis motility (when pegging is performed). In the realm of porous ocular grafts, hydroxyapatite (HAp) was the first material utilized (Suter et al., 2002). Because of the high biocompatibility of orbital tissues, minor exposures inclined to recover spontaneously. As a result, HAp spheres made from a cancellous bovine bone were employed in the early 20th century with good long-term results (Schmidt, 1906; Schmidt, 1910). The bovine HAp sphere was revived in the 1970s after being momentarily abandoned due to the introduction of polymeric implants (Molteno and ChB, 1991). This implant or graft is still in use these days and is known as the “Molteno M-Sphere” (Figure 6); however, its usage is restricted in comparison with other implants because of its expensive cost and certain worries regarding the fragility of the bovine inorganic phase (permeability more than 80% vol.), which also restricts the ability of pegging (Jordan et al., 2000a). The initial hydroxyapatite implant is well tolerated, and extrusion of the implant is uncommon. A retrospective examination of 357 patients reveals a 2.6% extrusion rate over a 10-year period. Subjective positive tolerance of 71.2% is consistent with worldwide research findings. The dynamic development of the newborn anopthalmus in response to the size growth of the orbita and the precise volume replenishment of the adult orbita are currently not possible with commercially available porous hydroxyapatite materials and will need more studies (Norda and Meyer-Rüsenberg, 2003).

### Composite Grafts

**FIGURE 7:**
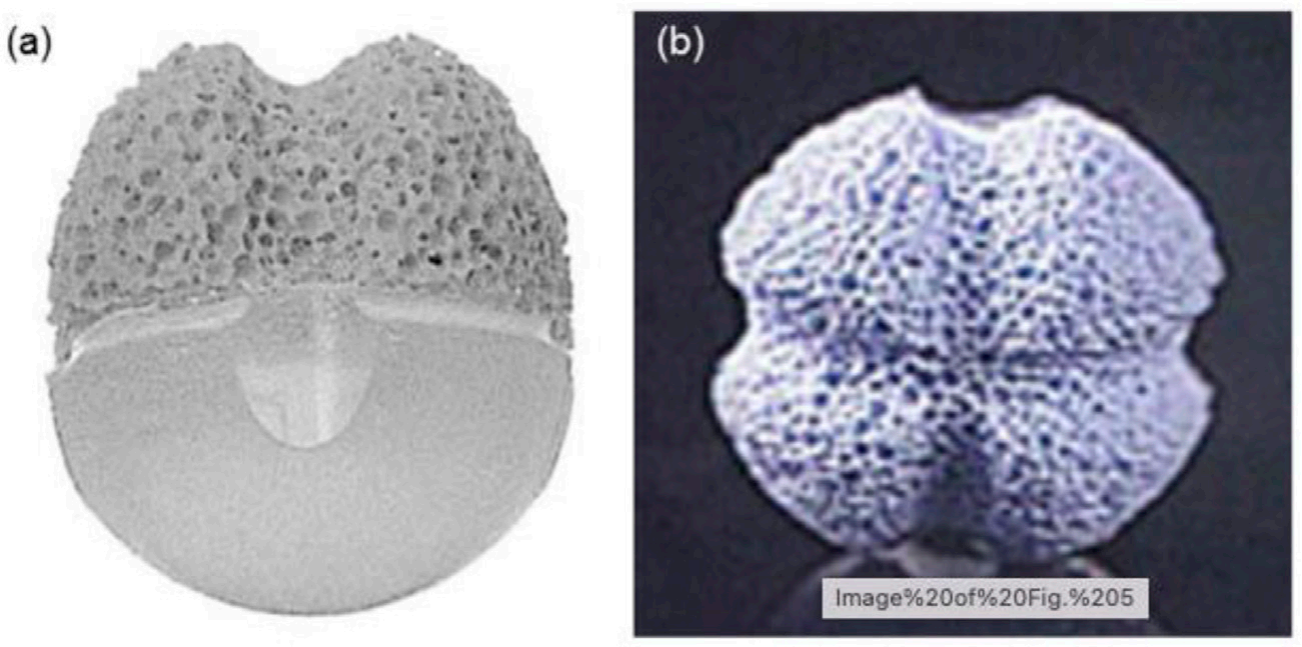
Illustration of the Guthoff implant: **(A)** lateral and **(B)** frontal views.

A previous study shows the multifaceted ingredients used in the production of OI. Between 1970 and 1990, two Teflon-based composite grafts, Proplast I (Teflon/carbon fiber sphere) (Neuhaus et al., 1984) and Proplast II (Teflon/alumina composite through a mount on the frontal side that might assimilate with the optical prosthesis in a “lock-and-key” fashion) (Girard et al., 1990a; Girard et al., 1990b), were tested in medical settings. Proplast II and Proplast I were later discontinued due to long-lasting adverse effects, such as late contaminations in the former (Whear et al., 1993) and deprived movability in the latter (Christenbury, 1991) due to lack of vascularization. Guthoff and others (Guthoff et al., 1995) established a composite implant in the 1980s that consisted of a frontal fragment made of artificial porous Hap for tissue incorporation and a subsequent silicone hemisphere/pinecone (Figure 7). The parallel and perpendicular eye muscles were stitched cross-sectional in front of the implant or graft to confirm better motility and stability.

In general, the biocompatibility of the graft was great, and the motility transfer to the visual prosthesis was excellent (Klett and Guthoff, 2003a; Klett and Guthoff, 2003b). This graft or implant is now commercialized and regarded as a great alternative, particularly in Europe; nevertheless, because of its expensive cost and more difficult surgical insertion method, its dissemination is restricted compared with “conventional” porous implants. Medpor-Plus OI, a mix of porous PE and 45S5 Bioglass^®^ particles (advertised in the trade name “Novabone” and frequently utilized as a bone grafting material) in a wt of 70 : 30% ratio, is a more modern type of composite device. Novabone was biocompatible when utilized with PE to increase the orbital volume in a rabbit model (Amato et al., 2003). In a research of 10 enucleated human patients, Naik et al. (Naik et al., 2007) compared the fibrovascular ingrowth of Medpor-Plus grafts to porous PE transplants as a whole (Medpor). MRI revealed a numerically substantial growth in the frequency of the fibro vascularization of PE grafts when utilizing the Novabone particle. One more study looked at the overall postoperative results of 170 patients who had a porous PE/bioactive glass composite implant placed following enucleation or secondary implantation and found a 94.7% overall success rate (Ma et al., 2011).

### Magnetic Implants

**FIGURE 8:**
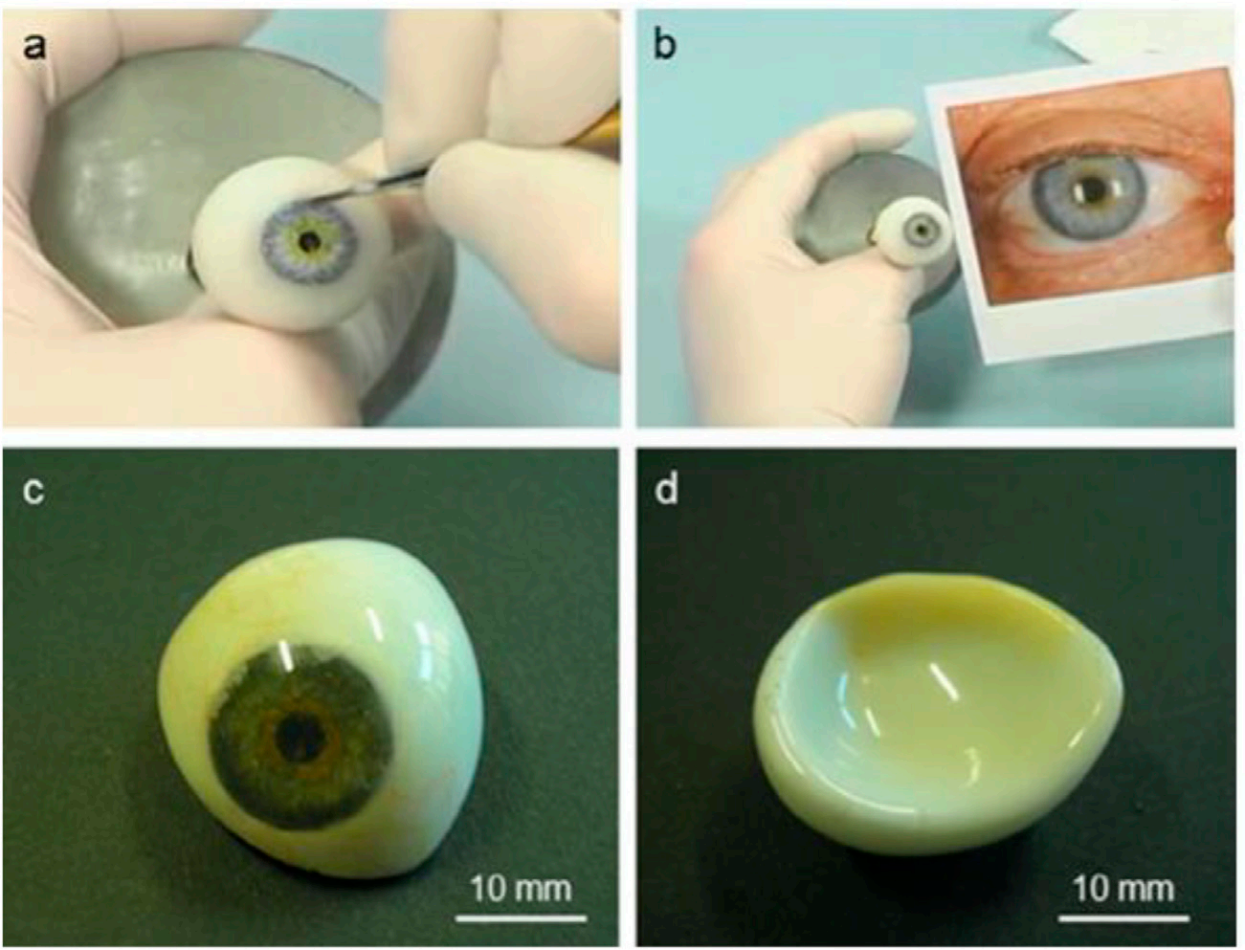
Example PMMA ocular prosthesis: **(A)** hand coloring of the iris button to match the aesthetic look of the healthy eye **(B)**, **(C)** frontal appearance of the finished prosthesis (with painted capillary vessels, iris, and pupil) following cleaning for optimum fit to the architecture of the client, and **(D)** backside convex surface.

**FIGURE 9:**
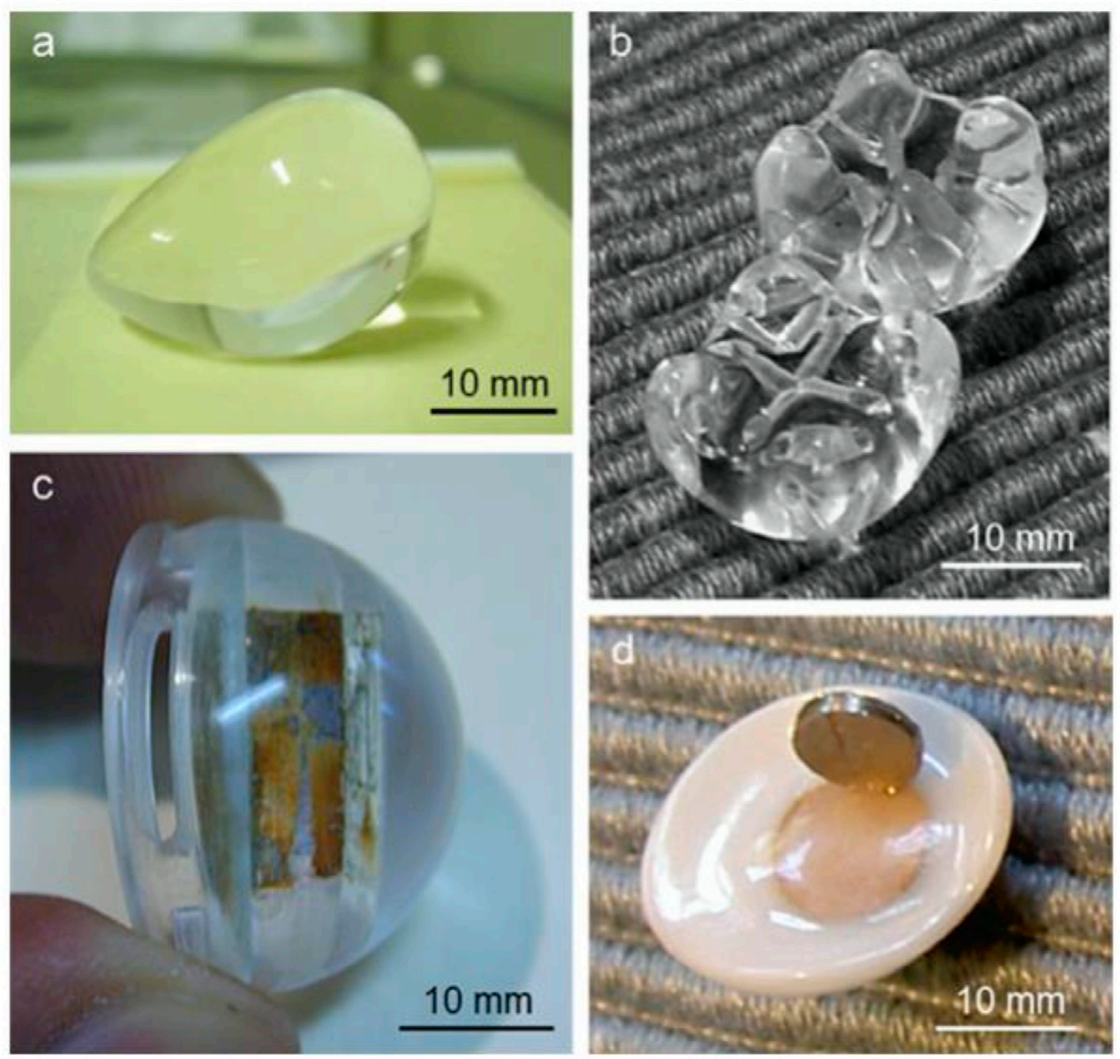
Utilization of polymethylmethacrylate (PMMA) for the fabrication of orbital implants: **(A)** a pear-shaped implant (Sahaf implant type I); **(B)** a comparison between the Iowa implant (upward) and the universal implant (downward), demonstrating that the latter has softer mounds in comparison with the Iowa predecessor; **(C)** a magnetic orbital implant; and **(D)** an associated ocular prosthesis that exhibits magnet rusting in both components. Reproduced with permission from (Sami et al., 2007).

One of the most pressing issues with the functional performance of the artificial eyes is how to retain the OP linked with the OI. Magnetic implants (MI), in comparison with other orbital devices, provide a “new” approach in this respect. The OP is maintained in place, and graft movement is communicated to it by the action of two magnets, one on the lateral side of the prosthesis and the other within the frontal area of the graft or implant, and the conjunctiva is squeezed in between the two parts. Following World War II, this technique was implemented, resulting in creating a variety of PMMA-based primary models influenced by the Allen-type sketch (Troutman, 1954; Young, 1954; Ellis and Levy, 1956; Roper-Hall, 1956; Myska and Roper-Hall, 1970; Atkins and Roper-Hall, 1983). The prosthetic eye was generally observed to move more horizontally than vertically, although this may be enhanced in both instructions if more magnetic bodies were put in the OP. Because these grafts were confined to a “conversational” range of movement, they did not have a large amplitude of movement. Ectropion and superior sulcus deformity were common complications, as was disclosure because of conjunctiva failure, which can happen when the magnet is excessively powerful or misplaced, producing aberrant firmness of the conjunctiva and Tenon’s capsule tissue among prosthesis and implant or graft (Soll, 1986). MIs have two seeming inevitable disadvantages while being a creative solution to the complication of implant–prosthesis integration (IPI). Sami et al. (2007) who identified confined toxicity caused by iron ion buildup inside the conjunctiva and accompanying tissue necrosis as two major reasons for conjunctival failure and late disclosure. Due to continuous interaction with biological fluids, PMMA absorbs water over time, causing magnet corroding with subsequent interaction along the center frontal surface opposite to the exterior margins, which are predisposed to pressure necrosis (Murray et al., 2000). The 2nd drawback applies to any metallic prostheses or grafts that may provide a risk during an MRI due to motion or displacement of the external metal item. Yuh et al. (1991) presented a situation of magnetic OIs extrusion triggered by 0.5 T MRI implant movement (Figures 8, 9).

## Discussion and Comparative Evaluation

### What, Where, and Why are the Chosen Materials and Implants?

Because each kind of implant has benefits and drawbacks, it is difficult to declare that one class of OIs is better to the others based on the available research; nevertheless, some suggestions can be provided. Many factors impact the selection of the “best” orbital implant, including the unique features of the damage, the clinical history and age of the patients and the experience and judgement of the surgeon. Furthermore, complicated oculoorbital surgery is often required in specific situations—e.g., when a midfacial shock has happened—and often involves both enucleations of the sick eye and repair of the broken orbital floor/wall (Dubois et al., 2015a; Dubois et al., 2015b). Mourits et al. (2015) conducted a computerized poll to determine the operational methods and grafts utilized for enucleation in retinoblastoma patients. The replies came from 58 surgeons operating in 32UK

countries worldwide. They analyzed these data to learn more about the materials utilized (Figure 6A) and discovered that the conventional PE sphere (19.2%) is the most frequent implant, pursued by PMMA ball (13.7%) and synthetic Hap (16.4%). Most surgeons favor porous implants directly above nonporous spheres, according to a study of aggregated data presented in Figure 6B (54.7% vs. 37.7%). It is worth noting that the % of porosity implants described in the research of Mourits et al. (Mourits et al., 2015) is in perfect accord with an approximation based on a questionnaire sent to United Kingdom ophthalmologists a decade previously (Viswanathan et al., 2007). The results of a prior assessment of a common medical practice in the care of the ophthalmic orifice revealed that porous OIs (PE, HAp, or alumina) were chosen in 55% of patients. In contrast, PMMA Allen-type implants were selected in 42%. New advancements [i.e., the Medpor SST (Choi et al., 2013) and Medpor QuadTMimplant (Young, 1954; Ellis and Levy, 1956)] continue to be made to porous implants, albeit at a greater cost. This is perhaps the main reason why, among the numerous types of porous grafts or implants currently on the retail shop, most surgeons still choose to use the “standard” simple spherical. In general, surgeons in Europe, the United States, and Canada (Adams et al., 2014) and in the Arabian States (Marx et al., 2008) favor porous maneuvers and Allen-type grafts. In a recent assessment of intraocular cancer therapy in the Asian pacific area, silicone or acrylic spheres were found to be the chosen implants (about 90%) in every patient categories (children, adults, and elderly) (Wang et al., 2014). It is worth noting that the PMMA solid scope is also the favored choice for 63% of oculoplastic doctors in Brazil. Further parts of the globe employ additional forms of implants for financial reasons, e.g., PMMA Sahaf implants are used by Pakistani surgeons because they are cheaper than Allen-type or porous orbital instruments supplied from other nations (Siddiqi et al., 2008). For example, Mourits et al. (Adams et al., 2014) point out that each surgeon has different reasons for utilizing certain materials and methods, such as implant availability, cost, expertise (theoretical) reappearance risk, and aesthetic result. Most hospitals appear to follow a procedure depending on the contract of surgeons operating in a similar center (Mourits et al., 2015), but there is no accepted international protocol.

### Are Porous Pmplants Better Than the Other Types?

Several surgeons recommend porous implants as a viable alternative for reducing the risks of exposure and extrusion. Furthermore, compared with Allen-type devices, PIs (and in common spherical maneuvers) need easier operational procedures and abilities. Exposures in porous devices, according to some authors, are more agreeable to conventional treatment without the need for a 2nd operational procedure, whereas disclosures in nonporous grafts or implants (i.e., universal Implant or acrylic sphere), if not very restricted, nearly always necessitate implant exclusion (Geirsdottir et al., 2014; Hauck and Steele, 2015). Porous implants have two significant benefits, according to theory (Hauck and Steele, 2015). 1) The implant is reduced to be expected to migrate or extrude, for the reason that fibrovascular tissue penetration during the extremely interrelated network of macropores (distinctive size 100–500 m) instinctively anchors the soft tissues of the orbit to the material and 2) vascular resource permits protected surveillance, which decreases postoperative contagions and stimulates curative of soft tissue nearby the graft. PIs, on the other hand, nevertheless, have an effective contact rate; the amount to which this is dependent on the biomaterial or other features, for example, operational procedure, is unclear (Cleres and Meyer-Rüsenberg, 2014; McElnea et al., 2014). In this context, McElnea et al. (McElnea et al., 2014) noted that the contact rate of porous OIs is considerably lesser when an orbital surgeon conducts an operation. However, postoperative issues are more probable when procedures are carried out by surgeons with the exterior of the subspeciality interest of oculoplastics.

### Use in Pediatric Inhabitants

The use of PIs in children is also up for discussion. Because of the upcoming volume extension to keep common bone/orbit progress and the eventual necessity for implant interchange with a bigger one, ease of exclusion would be addressed when treating youngsters. Due to the lack of fibrovascularization, nonporous grafts with a flat appearance, like, silicone and PMMA spheres, are simple to eliminate and are frequently favored by surgeons. On the other hand, few writers have documented a successful usage of porous instruments in youngsters (Shah et al., 2015). In a current research (531 instances of enucleation), HAp OIs in pediatrics patients had extremely good long-term results in relation to motility and patient/family aesthetic gratification (Shah et al., 2015), but future implant exchange issues were not explored.

### Pegging

Pegging is a technique for improving mobility and life-like look in PIs. Pegging can be done in PIs to increase motility and life-like form. OIs are typically enclosed anteriorly by the conjunctiva (“buried implants”) to segregate them from the peripheral environment. Jordan et al. (Jordan et al., 2016) have examined the current indications for pegging in depth. Pegging is conducted in a lesser of instances globally (5%–7%) (Viswanathan et al., 2007) because to added price and pressure to the patients, despite the substantial benefits that may be gained, particularly in improving horizontal motions (there will be a 2nd surgical operation required). The motility of simulated eyeballs in patients with unpegged (however, constantly enfolded) PIs, on the other hand, is comparable with that seen in patients with nonporous spherical complements (Custer et al., 1999). If pegging is not an option, the PMMA UIs, Medpor QuadTMMotility implant, and Guthoff device are excellent substitutes for porous spheres.

### Implant Salvage Exposure, Wrapping, and Procedures

The chemical composition and microstructural/physical characteristics of accessible implants vary considerably, and these differences may be to blame for the emergence of problems. The high “biocompatible” an implant is, the less inflammatory (quiescent) the eventual host reaction will be (Williams, 2008). Surface roughness, both macro and micro, is important in the progression of conjunctival weakening and consequent exposure/extrusion. Fine-grained (i.e., alumia) or smooth (i.e., PE, silicone, and PMMA) surfaces are preferable above coarse-grained materials (e.g., HAp), as rough surfaces should be abrasive to the nearby soft tissue when the implant travels (Xu et al., 1997). Direct interaction between the implant surface and the conjunctiva would be evaded, particularly when using PIs that are rough and stiff, like CIs. The implant might be put inside the sclera of the patient without extra draping if evisceration is performed; however, the implant should be coated (Gawdat and Ahmed, 2014). To aid vascularization of the porosity implant, the covering material (a thin layer of natural tissue or artificial polymer) would include disjointedness (holes). This kind of wrapping is essential since some materials might cause an inflammatory reaction, making them appear uncomplimentary and increasing disclosure (Rosner et al., 1992). Several enfolding materials perform worse than a simple graft, with Mersilene sheet exposure of 53% (related to 8% without draping) (Neuhaus et al., 1984) and polyurethane enfolding exposure of 46% (related to 5% of bare implant) [167]. Donor sclera has been the great often utilized draping material in the past, and it has been linked to a small intricacy rate (lower than 3%) [168, 169]. After the report of an incidence in the United Kingdom in 1997, where both the corneas and scleras from a donor, later found to have sporadic Creutzfeldt-Jakob disease, were transferred; the use of banked sclera has dropped (Tullo et al., 2006). Despite the fact that no disease transmission has been documented to date, this incidence prompted a revision in donor selection criteria; nowadays, scleral transmission risks are extremely minimal when donors are properly screened, and the tissue is handled according to procedure (Heimann et al., 2005). Table 1 summarizes the various implants along with their advantages and disadvantages.

**TABLE 1:** Various implants and materials with their advantages and drawbacks.

Type of material/implant	Advantages	Limitations/drawbacks
Allen-type implants (e.g., universal implant)	-promising motility	-require *ad hoc* fabricated ocular prosthesis fitting precisely the implant anterior
		-exposure result in requiring implant elimination
		-elaborate surgical installation
Coralline HA (porous)	-permit fibrovascularization	-pediatric patients are not eligible
	-promising motility (permit pegging)	-conjunctival abrasion risk
		-expensive
Porous alumina	-permit fibrovascularization	-expensive
		-pediatric patients are not eligible for it
	-promising motility (permit pegging)	
	-smooth surface than other porous materials	
Solid (nonporous) polymeric sphere (such as PMMA and silicon)	-simple technique	-fibrovascular ingrowth is not permitted
	-directly implantable	-exposure is less amenable of conservative than other porous materials
	-both pediatric and older patients are eligible	
AlphaSphere	-simple install orbit	-implant fragmentation after some time
	-direct implant suturing	
	-smooth surface	
	-permit fibrovascularization	
Guthoff implant	-permit fibrovascularization is allowed	-elaborate surgical procedure
	-promising motility	-expensive

## Surface Coating: A Significant Technique for Later-Generation Orbital Implants

The use of various surface coatings that can stimulate fibrovascularization or have an antibacterial impact is an intriguing technique that is being investigated to improve the achievement of OIs in comparison with the present state of the art. You et al. described the first effort to enhance vascularization by coating alumina implants (You et al., 2003). They placed a thin layer of man-made HAp on the grafts or implants. The goal of this technique was to make use of the load-bearing properties of alumina, however also utilizing the biocompatibility and long-lasting stability of HAp. The writer measured fibrovascularization in eviscerated rabbits following 2, 4, and 12 weeks after implantation and observed fibrovascularization at the implant periphery after 2 weeks and in the center after 4 weeks. Jordan et al. (2002) conducted a follow-up study on calcium phosphate coatings on porous alumina implants. According to their histological research, the coatings did not assist or prevent fibrovascular ingrowth in rabbits at 4, 8, or 12 weeks after implantation; hence, this technique was abandoned. Jin et al. (Jin et al., 2016) demonstrated efficacy in promoting fibrovascularization in porous HAp OIs using a biomimetic polymer covering. With five collagen/heparin multilayers, these investigators generated a layer-by-layer construction technique to alter the implant surface (Jin et al., 2016). The average pore size of the polymer-coated HAp scaffold remained acceptable for the anticipated application (approximately 316 m), and the mechanical strength was enhanced over the uncoated HAp device, according to SEM characterization (3.5 vs. 2.5 MPa). The elastic modulus dropped somewhat in interaction with soft tissues, which is ideal. After 14 days of culture, the polymer-coated HAp implant stemmed in greater cell propagation in an *in vitro* experiment utilizing human umbilical vein endothelial cells. The *in vivo* angiogenic potential of the implants was further assessed using a chicken chorioallantoic membrane (CAM) test. The CAM assay revealed that polymer-coated scaffolds had more intensive.

Neovascularization then mention implants based on macroscopic assessment and semiquantitative vascular density measurement. In addition, the same investigation team tested these scaffolds *in vitro* with mesenchymal stem cells and *in vivo* with a basic animal model (hypodermic pocket in rats) and set up a considerably greater density of freshly designed vessels and appearance of endothelial distinction indicators than the control group (Jin et al., 2016).

## Methodological Remarks—Future Research on Orbital Implants

This segment compiles various methodological remarks to provoke debate amongst academics and offer valuable recommendations for improving more operative OIs.

### Remarks on Implant Fabrication and Material Selection

The type of substance utilized as an OIs and its fate must be carefully examined. Because orbital implants must function as persistent instruments for filling the socket volume and maintaining the orbital tissues above the course of the lifetime of the patient, *in vivo* resorption should be prevented.

As a result, materials like soluble Ca_3_(PO4)_2_ (i.e., - and - TCP), bioresorbable polymers [i.e., poly (glycolic acid) (Gentile et al., 2014), and phosphate glasses (Colquhoun and Tanner, 2015)], while encouraging for other operational uses, must be thrown away or handled with extreme caution. Durette proposed using soluble bioactive glasses to make certain portions of investigational OIs that would progressively improve its porosity after being partly absorb again *in vivo*, allowing better entrance to blood arteries and fibrovascular tissue. However, partial resorption raises serious issues regarding the mechanical stability of the implant in excess of time. To the best of our information, this technique is no more being followed (no new investigations have been described in the papers to date) and keep a patented concept. Bioactive glasses have piqued the interest of ocular biomaterial investigators in recent years, and some have been used to make OIs (porous spheres (Xu et al., 1997) or as coatings on preexisting substrates (Ye et al., 2014) or solid cones). Although bioactive glasses display a potential in this sector, they would be used with caution as OI materials. First, a bioactive glass structure must be created by cautiously determining the quantity and ratio of the various fundamental oxides: this is an important step since glass formulation has a significant impact on the physicochemical and biological characteristics of the final material. The available literature (Wilson et al., 1981) provides useful information regarding the compositional boundaries of the bioactive glasses (no biological bond, bond to soft tissues, and bond to hard tissues). Aside from the benefit of attaching to delicate orbital tissues, bioactive glass OIs may be little costly than other bioceramics like HAp and alumina because of reduced processing temperatures and time. Small quantities of other metal oxides should be added to the glass formulation to fine tune its bioactive characteristics and cause the release of suitable ionic sorts, like, Cu2+, which has been shown to have antibacterial activities (Ye et al., 2014).

Dermal replacements are advantageous in situations when the standard surgical method is insufficient for any reason. Because they are infrequently employed in the periocular area, there is a dearth of literature on the subject. Nonetheless, based on available case reports and limited series, we may infer that the use of dermal replacements in the periocular area is often effective and free of problems (Kopecký et al., 2021).

### Observations on the Characterization and Testing of Materials

Once novel OIs have been created, it is critical to ensure suitability for the desired function. There is currently no well-defined, widely accepted, and rigorous methodology for evaluating novel orbital implants. As previously mentioned (Baino, 2011a), this is a typical issue with other ocular implants. Based on the appropriate literature and the knowledge of the authors, certain recommendations are made here to spark conversation among academics functioning in the subject. A solubility test in a suitable medium and *in vitro* testing of biological compatibility with cells would be the 1st two exclusion conditions for material selection. If bioactive implants are put to the test, ion release kinetics would be sensibly evaluated; meanwhile, materials that cause nonphysiological pH changes or ion release patterns that might be harmful to ocular tissues should be avoided. If the OIs contain medicines or growth factors, the release kinetics of the biomolecules would be tracked. The standard testing medium must be specified, considering that they should, at the very least, simulate the physiological milieu in which the orbital implant will be implanted.

### Is There a Way to Make Implant Development More “Global”?

As mentioned in *Remarks on Implant Fabrication and Material Selection and Observations on the Characterization and Testing of Materials*, a range of parameters linked to the materials utilized and graft style impact OIs performance containing crystalline phase presence, size/shape, surface roughness, and mechanical qualities if the graft is porous, pore features. It is not easy to consider the influence and significance of all of these variables. As a result, outlining a quantifiable and objective limitation “selection score” might aid surgeons in selecting and biomaterials researchers in developing more successful and quite customized OIs. Moreover, practical usage of such a “global” parameter might made OI selection less random and less reliant on the abilities and personal experience of the ophthalmic surgeon. To date, in tissue engineering, a quantitative criterion has been suggested to evaluate the achievement of scientific scaffolds to that of the bone tissue they are supposed to exchange. The operational and automated assets of two marketable synthetic implants to the trabecular bone were compared by Falvo D'Urso Labate et al. (2016) and created a quantitative measure to assess how closely the scaffold resembles real tissue and, therefore, whether it is a good contestant for bone grafting.

## Current Patents on Ocular Implants, Ranging From Research to Therapeutic Use

Many years ago, the most frequently utilized OIs (such as porous HAp, Allen-type, alumina, and PE) were developed and there have been very few new patents submitted in the recent decade (Jordan et al., 2000b). Patents offer the possibility for translating study findings into biological goods, and they are essential for meeting the unmet clinical requirements of the patients. But, none of the devices has been approved for medical use, and no research on them has been published in the scientific literature. On the contrary, encouraging results concerning certain other nonpatented new implants [e.g., OIs with a bioactive covering (190–195)] have been published. There is a gap among technical developments and medical applications, as Fernandez-Moure (Ye et al., 2014) pointed out, for various reasons. Patented inventions are seldom turned into FDA-accepted instruments, and still fewer are widely embraced by the clinical community (Bagchi-Sen, 2007). Certain of the maneuvers show very minor differences or claimed enhancements compared with “parent” implants, implying that firms are continuing to reutilize “old” tools to speed up the FDA clearance method and fulfil their economic targets. New materials, on the other hand, are still being developed and tested. However, their clinical influence is minimal since many physicians depend on a small number of expedients for the proposed operational use. Finally, it must be overlooked that, to preserve financing and professional progress, academics are sometimes pushed to forgo time and research-intensive translatable investigation to stress publication-making work. In conclusion, while new prospects happen in the area of OIs for emerging novel maneuvers with higher achievement, there is a gap in transmitting these study achievements to therapeutic applications, i.e., advancements at the bedside do not necessarily match to developments on the seat (Fernandez-Moure, 2016). Maybe, as Fernandez-Moure (2016) suggests, we could recognize that the trip initiates and finishes at the bedside and hold a new paradigm of translatable investigation that takes us to the bedside to the seat and back, redefining interpretation.

Moreover, there is a dearth of established treatment protocols for managing discharge. Frequent prosthesis removal and cleaning were related to more severe discharge, but the cause-and-effect relationship was not established. Professional repolishing regimens had a negligible effect on the discharge experience. Additional study on the response of the socket to prosthetic eye use is suggested, focusing on the physical, chemical, and biological components of the conjunctiva, socket fluids, and the deposits that coat the prosthetic eye (Pine et al., 2012).

## Conclusion and Summary

The functional evolution of anophthalmic socket surgery biomaterials and implants can be directly linked to their historical history. As a result, the history of orbital implants may be split into four primary eras, each with its unique set of characteristics: 1) the period of nonporous spherical grafts, where the key goal was to exchange the socket volume with a harmless material; 2) the age of Allen-type grafts, where the chief goal was to ensure good motility to the Ops; 3) the time of PIs, where the leading goal was to advance fibrovascularization; and 4) the era of porous implants, where the main goal was to ensure the current age of smart, multifunctional implants, in which the goal is to provide crucial additional benefits to the implant, like, *in situ* mold capability or antibacterial and angiogenetic characteristics. Biomaterials are openly requested to show a significant part in this fourth, upcoming age, although graft style was typically prioritized over material characteristics and functions in the past. Scientists are reporting surprising, smart characteristics of existing biomaterials in the literature, indicating that not only are new biomaterials being produced but also that scientists are reporting unexpected, smart qualities of existing biomaterials. For example, several bioceramic compositions have recently demonstrated the capacity to bind to soft tissue and promote angiogenesis, making these materials possibly appropriate for various soft tissue applications, containing eye operation that was previously unimaginable (Miguez-Pacheco et al., 2015). Observing at the current options, the advantage of PIs over nonporous grafts is debatable. Numerous research recommends that porous implants have a lower rate of implant extrusion and socket infection, which sustains the theory that vascular ingrowth anchors the graft and allows for resistant observation; however, comparison are difficult because of differences in surgical techniques, implant sizes, and follow-up periods.

Furthermore, there is a lot of room for surface modification on the orbital implant through the use of coatings that should stimulate a definite biological or beneficial retort at the graft–host tissue contact. Keeping in mind that one of the main goals is to enhance fibrovascularization, using bioactive composites or coatings that can release angiogenic mediators is a potential technique that should be investigated more in the upcoming ahead. Bioactive glasses are particularly appealing biomaterials in this context (Miguez-Pacheco et al., 2015), as ion dissolution products free from them have been shown to stimulate angiogenesis. This intriguing characteristic has mostly been used in the state of wound soothing and dressing, although Naik et al. (2007) describe increased angiogenesis in ocular grafts as well. Providing bFGF *in situ*, such as by enveloping the (porous) graft with a piece of bFGF-healed collagen, has also been demonstrated to improve fibrovascularization.
